# Trafficking and Activation of *Henipavirus*, *Parahenipavirus,* and Henipa-like Virus Fusion Proteins

**DOI:** 10.3390/v17060866

**Published:** 2025-06-19

**Authors:** Chanakha K. Navaratnarajah, Roberto Cattaneo

**Affiliations:** Department of Molecular Medicine, Mayo Clinic and Mayo Clinic School of Biomedical Sciences, Rochester, MN 55905, USA

**Keywords:** viral entry, zoonotic diseases, paramyxovirus, nipah virus, hendra virus, cedar virus

## Abstract

*Henipaviruses* are emerging zoonotic viruses that have caused deadly outbreaks in humans and livestock across several regions of the world. The fusion (F) protein of *henipaviruses* plays a critical role in viral entry into host cells and represents a key determinant of viral pathogenicity. This review provides a comprehensive analysis of current knowledge regarding trafficking, activation, as well as the role in particle assembly, of *henipavirus* F proteins. We discuss the unique characteristics of *henipavirus* F proteins compared to other paramyxovirus fusion proteins, with particular emphasis on their distinctive trafficking and activation mechanisms. Attention is also given to novel *henipaviruses* that have been detected in hosts other than bats, namely rodents and shrews. These viruses are sufficiently different that the International Committee on Taxonomy of Viruses has proposed a new genus for them, the *Parahenipaviruses*. We discuss how variations in F protein characteristics between *Henipaviruses*, *Parahenipaviruses*, and yet-unclassified henipa-like viruses might influence their trafficking and activation. Understanding these molecular mechanisms is crucial for developing effective therapeutic strategies against *henipavirus* infections and for predicting the emergence of novel *henipavirus* strains with pandemic potential.

## 1. Introduction

The first recorded *Henipavirus* zoonotic event occurred in 1994 in Australia following an outbreak of fatal cases of respiratory disease in horses and humans, leading to the discovery of Hendra virus (HeV) [1]. This was followed by a large-scale outbreak of a related virus in 1998 in pig farms in Malaysia, resulting in 283 symptomatic cases and 109 deaths (39% case fatality rate [CFR]). This led to the isolation of Nipah virus (NiV) in 1999 [2]. Together, HeV and NiV formed the basis for the *Henipavirus* (HNV) genus in the *Paramyxoviridae* family of negative-sense RNA viruses [3,4,5]. They cause severe and often fatal respiratory and/or neurologic disease in both humans and animals [2,6]. HeV continues to cause sporadic outbreaks in horses, which occasionally spill over into humans with a 57% CFR (4/7) [7]. Meanwhile, NiV spillover events have been occurring annually in South Asia since 2001 [8,9,10,11,12,13,14,15,16,17] and as of May 2024, 745 confirmed cases of NiV have resulted in 435 deaths in this region (58% CFR) [18].

**Figure 1:**
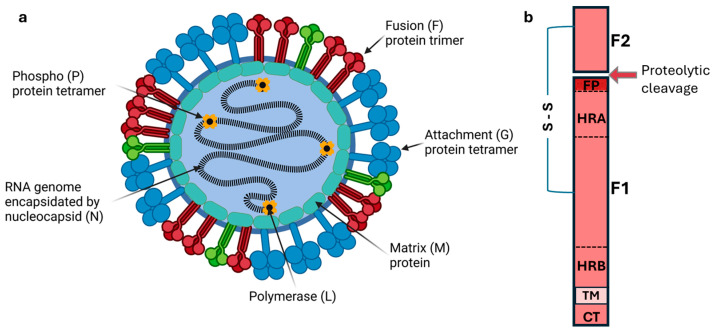
Schematic of *Henipavirus* particle and fusion protein. (**a**) The nucleocapsid (N) protein (black lines) encapsidates the negative-sense, single-stranded RNA genome. The RNA-dependent RNA polymerase (L, black dot) and phosphoprotein tetramers (P, yellow shapes) complete the replication complex. The matrix protein (M, green oblongs) interacts with the cytoplasmic tails of the fusion proteins (F, red or green trimers) and of the attachment proteins (G, blue tetramers) as well as with the N protein to direct particle assembly. Green trimers represent F proteins that have not been proteolytically processed while red trimers represent processed fusion-competent F proteins. (**b**) The *henipavirus* F protein is synthesized as an inactive precursor, which is cleaved into two disulfide bond-linked fragments, F_1_ and F_2_. This cleavage exposes a hydrophobic fusion peptide (FP) at the N-terminus of the membrane-anchored F_1_ fragment. The F_1_ fragment also contains two heptad repeats (HRA and HRB) that snap together into a six-helix bundle in the post-fusion state. The cytoplasmic tail (CT) contains signals that govern F protein trafficking. TM, transmembrane.

HNVs are enveloped, negative-sense, single-stranded RNA viruses with genomes of approximately 18–20 kb encoding six structural proteins: nucleocapsid (N), phosphoprotein (P), matrix protein (M), fusion protein (F), attachment glycoprotein (G), and large polymerase (L) [3] (Figure 1a). The entry of HNVs into host cells is mediated by two surface glycoproteins: the attachment glycoprotein (G) tetramers and the fusion protein (F) trimers [19]. The G protein is responsible for binding to host cell receptors, which, for HeV and NiV, are primarily ephrin-B2 and ephrin-B3 [20,21,22]. These are highly conserved proteins across mammalian species and contribute to the broad host range of *henipaviruses* [6]. The F protein, meanwhile, mediates the fusion of the viral and host cell membranes, allowing the viral ribonucleocapsid (RNP) to enter the cytoplasm and initiate infection.

**Figure 2:**
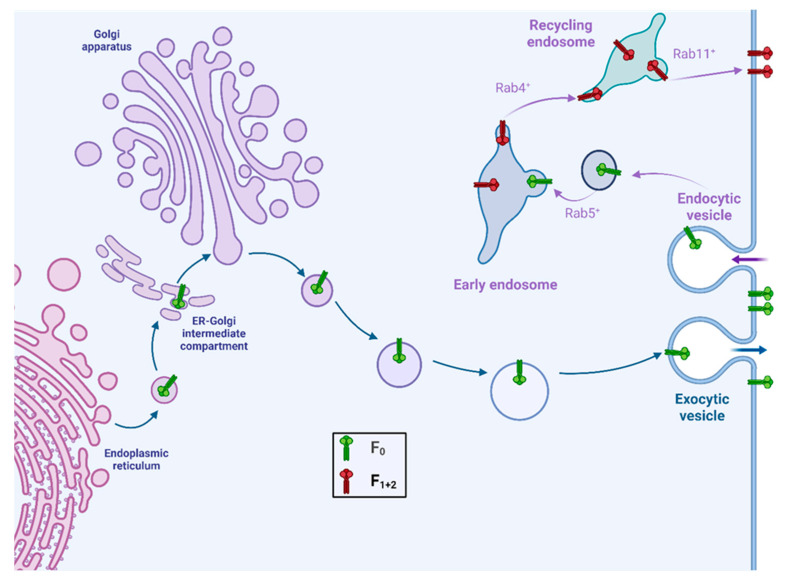
Trafficking and activation of the *henipavirus* F protein. From bottom left: The F-trimer is transported to the plasma membrane as the precursor F_0_ (green trimers, blue arrows). Upon endocytosis (purple arrows), F_0_ is trafficked to Rab5^+^/4^+^ early/sorting endosomes, where it is cleaved by endosome-resident proteases into F_1+2_ (red trimers). This fusion-competent F protein is returned to the plasma membrane via Rab11^+^-recycling endosomes. The F protein may interact with the attachment (G) protein and the matrix (M) protein at or near the plasma membrane to initiate particle assembly.

The HNV F protein distinguishes itself from the F proteins of other paramyxoviruses through several unique characteristics related to its trafficking and activation. Like most paramyxovirus F proteins, the HNV F protein is synthesized as an inactive precursor, F_0_ (Figure 1b). However, unlike most paramyxovirus F proteins, which are activated by cellular proteases prior to reaching the cell surface, *henipavirus* F proteins reach the surface in its inactive form (Figure 2, green F-trimers). Endocytic signals in the cytoplasmic tail direct the uptake of the F protein into endosomal compartments where endosome-resident cathepsin proteases cleave it into disulfide bond-linked F_1_ and F_2_ fragments [23,24,25,26,27,28] (Figure 1b and Figure 2). The fusion-competent F_1+2_ protein is then returned to the cell surface for particle assembly. This distinctive activation mechanism represents an intriguing adaptation that may influence the tissue tropism and pathogenicity of HNVs.

Understanding the molecular mechanisms underlying HNV F protein function is crucial for developing effective therapeutic strategies and for predicting the emergence of novel *henipavirus* strains with pandemic potential. Furthermore, insights into the unique activation mechanism of *henipavirus* F proteins may have broader implications for our understanding of viral entry mechanisms and the evolution of viral pathogenicity. Thus, the purpose of this review is to provide a comprehensive analysis of current knowledge regarding the distinctive trafficking and activation mechanisms of the F proteins of *Henipaviruses*, of the recently discovered *Parahenipaviruses*, and of the yet-unclassified henipa-like viruses.

## 2. Natural Reservoirs and Taxonomy of *Henipaviruses*, *Parahenipaviruses*, and Henipa-like Viruses

Bats are the reservoir for HeV and NiV [8,29], specifically *Pteropus* spp. fruit bats whose range extends as far west as Madagascar, through the Indian subcontinent to Southeast Asia and Australia, and eastwards through Oceania [30]. In fact, the entire *Henipavirus* genus exhibits a predominant association with bats as their natural reservoirs. According to the International Committee on Taxonomy of Viruses (ICTV), this genus encompasses five species [31] (Table 1). In addition to HeV and NiV, Ghanaian bat virus (GhV; formerly known as the Kumasi virus) RNA was detected in pooled fecal samples belonging to African straw-colored fruit bats (*Eidolon helvum*) located in Kumasi, Ghana [32]. Cedar virus (CedV) was isolated from the urine of *Pteropus* spp. fruit bats in Australia in 2012 [33]. Challenge studies in ferrets and guinea pigs, which are susceptible to *henipavirus* infection and disease, confirmed CedV replication and production of antibodies without apparent disease. This non-pathogenic phenotype is attributed to an impaired innate immune evasion capacity [33,34,35,36,37]. Finally, Angavokely virus (AngV) RNA was identified in Madagascar from a urine sample obtained from a Malagasy fruit bat (*Eidolon dupreanum*) in 2019 [38]. This strong association with fruit bats suggests a long history of co-evolution between these viruses and their bat hosts, potentially resulting in unique mechanisms that allow for viral persistence and transmission within these hosts without causing significant disease. Bats are well known for serving as reservoirs for a wide array of viruses, and their distinctive physiological traits, such as their ability to fly, their high metabolic rates, and their specialized immune systems, may contribute to their capacity to harbor and transmit numerous viral pathogens [39].

Currently, only three bona fide *Henipaviruses* (HeV, NiV and CedV) have been isolated, and two have been inferred by sequence homology (GhV and AngV). In recent years, advances in molecular surveillance and next-generation sequencing technologies have led to the discovery of additional henipa-like viruses, expanding our understanding of the genetic diversity and host range of these viruses [30]. Specifically, there is a growing appreciation of the significant role of shrews and rodents as natural reservoirs for henipa-like viruses. Mòjiāng virus (MojV) was the first HNV to be discovered in a natural reservoir other than bats. The MojV genome was identified in cave rats in China after the death of three miners from pneumonia [40]. Gamak virus (GAKV) and Daeryong virus (DARV) were detected during small animal surveillance in the Republic of Korea between 2017 and 2018, with GAKV subsequently isolated from kidney tissue homogenates [41]. These viruses were identified in the mammalian shrew species *Crocidura lasiura* and *Crocidura shantungensis*, respectively. Langya virus (LayV) was first identified in and isolated from febrile patients in the eastern provinces of Henan and Shandong in China [42]. Subsequent investigations revealed that wild shrews were a significant reservoir for this virus, with LayV viral RNA detected in a high proportion of the shrews tested. Camp Hill virus (Table 1, [CHV]), recently identified in Northern short-tailed shrews, is the first henipa-like virus discovered in North America [43]. Wild animal surveillance continues to uncover new henipa-like viruses in shrew and rodent reservoirs (Table 1). The discovery of henipa-like viruses in these small mammals, which exhibit a widespread global distribution and inhabit diverse ecological niches, suggests potential for these viruses to emerge in various environments across the world. The febrile illness caused by LayV and the suspected deaths caused by MojV are examples of zoonotic events caused by these shrew/rodent-borne viruses. However, for now only LayV and GAKV have been propagated in cultivated cells, which may imply restricted tropism.

**Figure 3:**
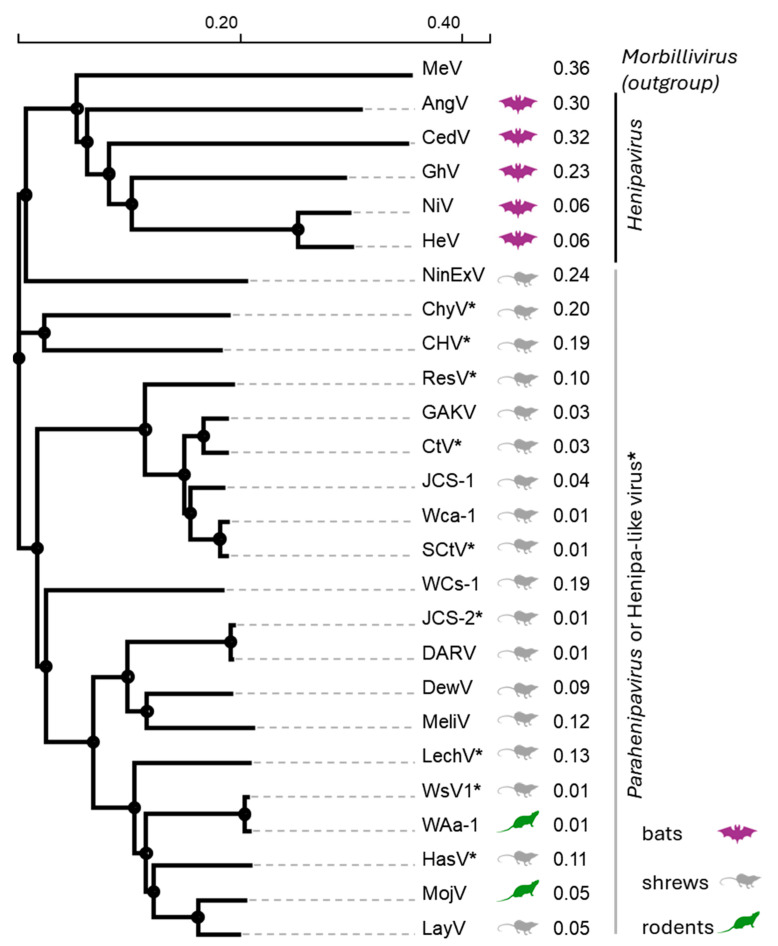
Phylogenetic relationships of *Henipaviruses*, *Parahenipaviruses*, and henipa-like viruses based on their F proteins. Amino acid sequences corresponding to putative F proteins were aligned using ClustalOMEGA (Version 1.2.2) [46], with the resulting phylogenetic tree generated using the Neighbor-Joining method. The optimal tree is shown. The percentage of replicate trees in which the associated taxa clustered together in the bootstrap test (1000 replicates) are shown next to the branches. GenBank accession numbers are indicated in Table 1. Viruses marked with a * have not been formally classified and are considered henipa-like viruses. Measles virus (MeV, NP_056922.1), Angavokely virus (AngV), Cedar virus (CedV), Ghana virus (GhV), Nipah virus (NiV), Hendra virus (HeV), Ninorex virus (NinExV), Chodsigoa hypsibia henipavirus (ChyV), Camp Hill virus (CHV), Resua virus (ResV), Gamak virus (GAKV), Crocidura tanakae henipavirus (CtV), Jingmen Crocidura shantungensis henipavirus 1 (JCS-1), Wufeng Crocidura attenuata henipavirus 1 (Wca-1), Shiyan Crocidura tanakae henipavirus (SCtV), Wufeng Chodsigoa smithii henipavirus 1 (WCs-1), Jingmen Crocidura shantungensis henipavirus 2 (JCS-2), Daeryong virus (DARV), Denwin virus (DewV), Melian virus (MelV), Lechcodon virus (LechV), Wenzhou shrew henipavirus 1 (WsV1), Wenzhou Apodemus agrarius henipavirus 1 (WAa-1), Hasua virus (HasV), Mojiang virus (MojV), and Langya virus (LayV).

Phylogenetic analyses based on the L gene of bat-borne and rodent/shrew-borne HNVs show a clear dichotomy between viruses that circulate in flying (bats) vs. nonflying (rodents/shrews) mammals [7,44]. Our phylogenetic analysis based on HNV F proteins also led to a similar conclusion: the bat-borne HNVs (AngV, CedV, GhV, HeV, and NiV) cluster closely together and are distinct from the rodent/shrew-borne viruses (Figure 3). In recognition of this clear taxonomic difference between the bat-borne and rodent/shrew borne HNVs, the ICTV in 2023 proposed to classify the latter group into a separate genus, the *Parahenipaviruses* [45]. In our phylogenetic analysis, the measles virus (MeV) F protein, which was included as an outgroup, was more closely related to the bat-borne HNVs than the bat-borne HNVs were to the rodent/shrew-borne HNVs (Figure 3). It is interesting to note that, initially, the disease caused by HeV was described as “a novel morbillivirus pneumonia of horses” [1]. Our phylogenetic analyses also indicate that AngV may be ancestral to the other four bat *Henipaviruses* since it is the earliest node within this genus (Figure 3, HeV, NiV, CedV, and GhV). The unassigned henipa-like viruses all cluster in the *Parahenipavirus* clades, suggesting that they may eventually be assigned to this genus. Finally, in view of the above, it behooves the ICTV to reconsider the genera within the *Paramyxoviridae* family.

There are several differences between the bat and shrew HNVs, including their genome organization. While the genome of *Henipaviruses* ranges from 16.7 to 18.2 kb, the genomes of *Parahenipaviruses* are longer, ranging from 18.4 to 19.9 kb [7]. The *Parahenipaviruses* encode an additional gene that is not found in bat-borne HNVs [47]. This gene codes for a putative, conserved transmembrane protein termed S, a little over 100 amino acids in size [48]. It is located within the 5′ untranslated region of the F gene and it is unclear if and how it is expressed as it does not have its own start and stop signals [49]. On the other hand, Ninorex virus (NinExV), detected in shrews in Belgium encodes a gene X of unknown function, which does have its own start and stop signals, located between the M and F genes [48]. However, no other *Henipaviruses* or *Parahenipaviruses* have been shown to encode this gene [50].

## 3. Glycoprotein Organization in the HNV Particle

The major class of *Henipavirus* particles is about 500 nm in diameter [2], although, as in other paramyxoviruses, large particles are detected (up to 1900 nm) [51,52]. The particles tend to be mostly spherical with a lipid envelope derived from the infected cell plasma membrane. Based on super-resolution microscopy work on HNVs and cryo-electron microscopy (cryoEM) and tomography studies of related paramyxoviruses, the lipid envelope is densely packed with F and G clusters [53,54]. The glycoproteins form clusters of F-trimers and G-tetramers that protrude from the lipid envelope (Figure 1a). Both the fusion-competent F_1+2_ (Figure 1a, red trimers) and the inactive F_0_ (Figure 1a, green trimers) can be incorporated into the particles [54,55]. The M protein acts as a bridge between the RNP and the glycoproteins (Figure 1a). While both glycoproteins can individually drive virus-like particle (VLP) budding, the M protein is critical for efficient particle budding and particle stability [56].

## 4. Fusion Protein Trafficking and Proteolytic Cleavage: *Henipaviruses*

Trafficking of HeV [24] and NiV [25,28] F proteins has been well characterized. More recently, CedV F protein analysis [57] further complemented this work. The HNV F protein is a class I viral fusion protein synthesized as an inactive precursor (F_0_) with an N-terminal signal peptide, an ectodomain, a transmembrane domain (TM), and a C-terminal cytoplasmic tail (CT) (Figure 1b). The ectodomain contains several functional regions, including the fusion peptide (FP), which is initially buried within the protein structure, two heptad repeat regions (HRA and HRB) that form coiled-coil structures during the fusion process, and multiple N-linked glycosylation sites.

Proteolytic cleavage of F_0_ generates two subunits: the membrane-distal F_2_ subunit and the membrane-anchored F_1_ subunit, which remain covalently linked by disulfide bonds (Figure 1b). Cleavage exposes the FP at the N-terminus of the F_1_ subunit, which is essential for membrane fusion activity. Receptor binding of the attachment protein G induces conformational changes, which in turn transmit a triggering signal to the F protein. This signal leads to conformational rearrangement of the F protein, exposing the hydrophobic FP. The FP, normally buried in a pocket in the prefusion state, inserts into the host cell membrane. Subsequently, the F protein undergoes a dramatic re-folding from its metastable prefusion form to a more stable six-helix bundle conformation, leading to the merger of the viral and host cell membranes [58,59].

HeV and NiV F proteins lack the polybasic furin protease cleavage consensus motif shared by many of the paramyxovirus F proteins [55]. Studies characterizing the requirements for proteolytic processing of the HeV and NiV F protein determined that the process is insensitive to decrease in cellular Ca^2+^ levels, but sensitive to increases in intracellular pH [25,60]. Moreover, the efficient replication of HeV in a furin-deficient cell line further confirmed that *Henipavirus* F proteins are not cleaved by furin. Initial studies using various protease inhibitors showed that inhibitors of cysteine proteases decreased HeV and NiV F cleavage [23,26]. Specifically, membrane-permeant cysteine protease inhibitors like E-64d and calpeptin inhibited HeV F cleavage. Crucially, a general cathepsin inhibitor (cathepsin inhibitor I) and specific inhibitors of cathepsin L (cathepsin L inhibitor III) and cathepsin B/L (CA-074Me) interfered with HeV F cleavage [23]. These specific and nonspecific cathepsin inhibitors also effectively abolished proteolytic processing of NiV F [26]. The inhibition profiles indicates that cathepsin proteases are involved in the maturation of HeV and NiV F proteins and the low pH requirement [25,60] aligns with cathepsin L localization in the endosomal/lysosomal pathway [61].

These results were corroborated by experiments using small hairpin RNA (shRNA) oligonucleotides designed to target cathepsin L [23]. These shRNAs strongly reduced cathepsin L expression and enzyme activity in Vero cells with a concomitant decrease in HeV and NiV F protein cleavage in a dose-dependent manner [23,26]. As expected, this inhibition of F proteolytic processing correlated with significant reductions in the protein’s ability to mediate cell–cell fusion. Mouse embryonic fibroblasts (MEFs) derived from cathepsin L^−/−^ (deficient) mice were used to further analyze the role of cathepsin L in NiV F cleavage [26]. While cell–cell fusion mediated by NiV F and G proteins occurred efficiently in cathepsin L^+/+^ (wild type) MEFs, expression of both NiV F and G did not lead to fusion in MEFs lacking cathepsin L. Importantly, cell–cell fusion activity was partially restored upon expression of cathepsin L with NiV F and G in the cathepsin L^−/−^ MEFs.

To confirm the ability of cathepsin L to directly process HeV and NiV F proteins, purified human cathepsin L was used to digest immunopurified uncleaved F_0_ protein in vitro [23,26]. Incubation of F_0_ with purified cathepsin L resulted in the proteolytic processing of F_0_ into products that corresponded to the F_1_ and F_2_ subunits. Interestingly, purified cathepsin B could also cleave NiV F_0_ into two fragments in vitro [26]. However, the smaller F_2_ fragment produced by cathepsin B migrated slower compared to the F_2_ subunit generated by cleavage in cells. This suggests that while cathepsin B can cleave F_0_ in vitro, it may not cleave at the correct site to produce the mature, fusion-active form of NiV F. On the other hand, some studies suggest that cathepsin B can also contribute to or mediate NiV F cleavage in specific cell types, such as MDCK cells [62]. To date, only the cellular proteases that cleave the F proteins of HeV and NiV have been identified. The authors have unpublished data indicating that the general cysteine protease inhibitor (E-64d) and the specific cathepsin L inhibitors (cathepsin L inhibitor III) block CedV F protein-mediated cell–cell fusion, suggesting that the CedV F protein may also be cleaved by endosomal cathepsins.

**Figure 4:**
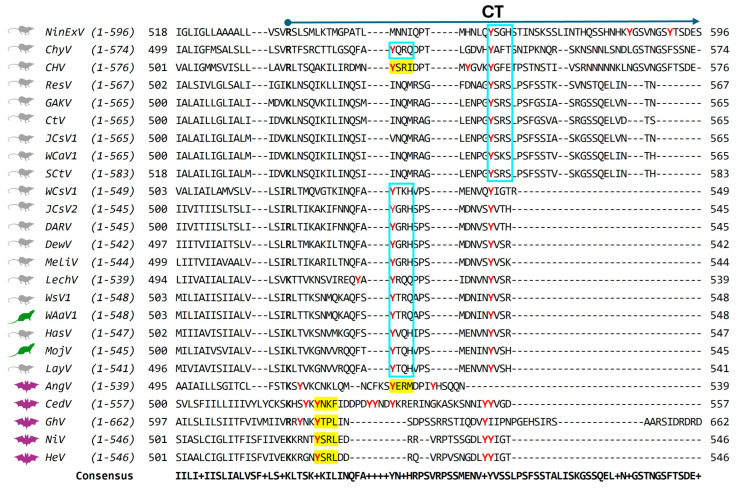
Tyr-based endocytic motifs and putative endocytic signals in the HNV F cytoplasmic tail. Sequence alignment of *henipavirus* F-CT residues was performed using ClustalOMEGA [46]. All Tyr residues are highlighted in red. Location of canonical endocytic motifs of the form YXXØ (where X is any amino acid and Ø is an amino acid with a bulky hydrophobic sidechain) is indicated by yellow highlights. Putative, non-canonical, Tyr-based endocytic signals are indicated with a blue box. CT, cytoplasmic tail. The first charged residue, which signals the end of the transmembrane region, is indicated in bold font. Black-dash lines indicate regions lacking alignment. Bat, rodent, and shrew reservoirs are denoted by the same symbols as in Figure 3. GenBank accession numbers are indicated in Table 1. Ninorex virus (NinExV), Chodsigoa hypsibia henipavirus (ChyV), Camp Hill virus (CHV), Resua virus (ResV), Gamak virus (GAKV), Crocidura tanakae henipavirus (CtV), Jingmen Crocidura shantungensis henipavirus 1 (JCS-1), Wufeng Crocidura attenuata henipavirus 1 (Wca-1), Shiyan Crocidura tanakae henipavirus (SCtV), Wufeng Chodsigoa smithii henipavirus 1 (WCs-1), Jingmen Crocidura shantungensis henipavirus 2 (JCS-2), Daeryong virus (DARV), Denwin virus (DewV), Melian virus (MelV), Lechcodon virus (LechV), Wenzhou shrew henipavirus 1 (WsV1), Wenzhou Apodemus agrarius henipavirus 1 (WAa-1), Hasua virus (HasV), Mojiang virus (MojV), Langya virus (LayV), Angavokely virus (AngV), Cedar virus (CedV), Ghana virus (GhV), Nipah virus (NiV), and Hendra virus (HeV).

Thus, unlike other *paramyxovirus* F proteins, *henipavirus* F proteins reach the cell surface as uncleaved F_0_ precursors (Figure 2). Cleavage occurs after re-internalization for proteolytic activation in the early endosome. Subsequently, the F protein returns to the plasma membrane for particle assembly and cell–cell fusion. Re-internalization of HeV, NiV, and CedV F proteins is driven by canonical tyrosine-based endocytic signals in the F protein cytoplasmic tail (CT) [24,28,57]. The tyrosine-based internalization signals of the form YXXØ, where X represents any amino acid and Ø represents an amino acid with a bulky, hydrophobic sidechain, are generally recognized by cytosolic adaptor complexes such as AP-2. These complexes selectively concentrate proteins within clathrin-coated vesicles for endocytosis and regulate further transport within the endosomal system. Consequently, mutagenesis of the HeV and NiV endocytic motifs (Figure 4, YSRL) greatly reduced endocytosis [24,28]. Similarly, mutagenesis of the canonical endocytic motif in the CedV F-CT (Figure 4, YNKF) resulted in a decrease in F internalization [57]. The F proteins of these three viruses also encode di-tyrosine (di-Tyr) motifs close to the end of the CT (Figure 4, red di-Tyr residues in bold type). Mutation of these motifs result in marginal reduction in endocytic rates and, in the case of CedV, an increased accumulation of F protein at the cell surface. The di-Tyr motifs also play a role in NiV F sorting in polarized endothelial [63], epithelial [64], and neuronal cells [65]. Mutation of all potential Tyr-based endocytic signals in NiV [28] and in CedV F-CT [57] completely blocked endocytic trafficking, indicating that multiple canonical and non-canonical Tyr-based endocytic signals govern HNV F trafficking.

Mutagenesis of the endocytic motifs in the cytoplasmic tail of HeV, NiV, and CedV F proteins had significant and sometimes unexpected impacts on fusion function. Mutation of Y525 resulted in a reduced rate of endocytosis for HeV F (Y525A) compared to the wild-type F protein, with a concomitant reduction in the rate of proteolytic processing [24]. Despite the reduced cleavage, HeV F Y525A was hyper-fusogenic, promoting cell–cell fusion more efficiently than the wild-type F protein. A similar hyper-fusogneic phenotype was noted for the corresponding mutation in the CedV F protein [57]. On the other hand, the same mutation in NiV F resulted in a hypo-fusogenic phenotype [28]. These findings highlight that while endocytic trafficking is crucial for the activation and function of HNV F proteins, the specific outcome of disrupting endocytosis via motif mutation can vary between these closely related viruses. One possible explanation for the hyper-fusogenic phenotype is accumulation of F protein at the cell surface because of the reduced endocytic rate. Indeed, the levels of surface expression for HeV and CedV F endocytic motif mutants were higher than that of wild-type proteins. While the ratio of fusion-active F_1+2_ to non-fusogenic F_0_ declined, the accumulation of more F protein at the cell surface over time leads to more efficient fusion. In the case of NiV F protein, we conjecture that the level of cell surface F accumulation may not be sufficient to overcome the deficit in the fusion-active form of the F protein, leading to a decline in fusion function.

**Figure 5:**
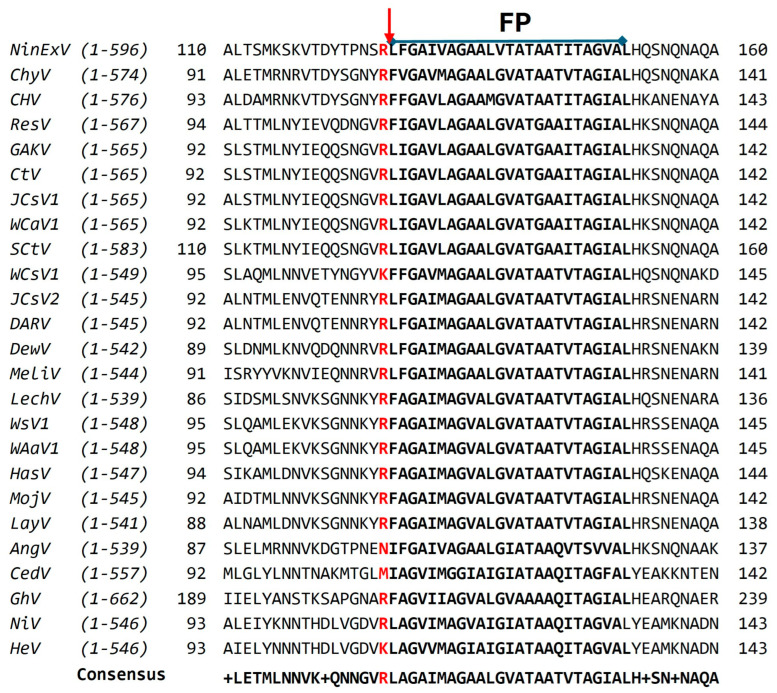
F1-F2 cleavage site of HeV and NiV and the predicted cleavage sites of the other *Henipaviruses*, *Parahenipaviruses*, and henipa-like viruses. Sequence alignment of HNV F protein residues was performed using ClustalOMEGA [46]. The residue after which cleavage occurs, or is predicted to occur, is indicated in red. Arrow indicates cleavage site. FP, fusion peptide. Known or predicted FP residues are indicated in bold font. GenBank accession numbers are indicated in Table 1. Ninorex virus (NinExV), Chodsigoa hypsibia henipavirus (ChyV), Camp Hill virus (CHV), Resua virus (ResV), Gamak virus (GAKV), Crocidura tanakae henipavirus (CtV), Jingmen Crocidura shantungensis henipavirus 1 (JCS-1), Wufeng Crocidura attenuata henipavirus 1 (Wca-1), Shiyan Crocidura tanakae henipavirus (SCtV), Wufeng Chodsigoa smithii henipavirus 1 (WCs-1), Jingmen Crocidura shantungensis henipavirus 2 (JCS-2), Daeryong virus (DARV), Denwin virus (DewV), Melian virus (MelV), Lechcodon virus (LechV), Wenzhou shrew henipavirus 1 (WsV1), Wenzhou Apodemus agrarius henipavirus 1 (WAa-1), Hasua virus (HasV), Mojiang virus (MojV), Langya virus (LayV), Angavokely virus (AngV), Cedar virus (CedV), Ghana virus (GhV), Nipah virus (NiV), and Hendra virus (HeV).

Little is known about F protein trafficking and processing of the two other *Henipaviruses*, GhV, and AngV. The entry receptor for GhV is ephrin-B2 (EFNB2) [66], as with the other *Henipaviruses*. While the GhV F protein is considerably larger than that of the other *Henipaviruses* (Figure 4, 660 amino acids vs. ~550), it does encode a canonical Tyr-based endocytic motif of the form YXXØ (Figure 4, YTPL). However, the functionality of this motif has not yet been experimentally determined. On the other hand, both proteolytically cleaved and uncleaved GhV F proteins reach the cell surface [67], suggesting that like other *Henpaviruses*, GhV F is also endocytosed for processing and then returned to the surface. While the predicted cleavage site (Figure 5, R205) fulfills the requirements for cleavage by cathepsin L [67], the amount of processed fusion-active GhV F at the cell surface was much lower compared to HeV and NiV F, which may contribute to reduced fusogenicity [67]. This observation hints at potential differences in the efficiency of proteolytic processing or the trafficking pathway that delivers the cleaved protein to the cell surface.

The entry receptor for AngV is yet unknown, and its G protein lacks the conserved ephrin-binding residues typically found in other *Henipavirus* G proteins, which is inconsistent with binding to cellular ephrins [38]. AngV F contains a canonical endocytic motif in its cytoplasmic tail (Figure 4, YERM), suggesting that it requires endocytosis for F activation. The predicted cleavage site (Figure 5, N103) is compatible with cathepsin L cleavage requirements [68].

In summary, all the bat-borne HNV species either have functional endocytic motifs or encode canonical endocytic motifs for potential processing by endosomal proteases such as cathepsin L. While only the HeV and NiV F proteins have been formally shown to be cleaved by cathepsin L and in some cases, cathepsin B, there is preliminary evidence that CedV F may also be cleaved by cathepsin L. However, there are differences in the endocytic rates and cleavage efficiencies of different *Henipavirus* F proteins, as evidenced by the varying amounts of fusion-active F that reaches the cell surface. These differences are also evident from the differential impacts of endocytosis motif mutations on the fusion function of different *Henipavirus* F proteins.

## 5. Fusion Protein Trafficking and Proteolytic Cleavage: *Parahenipaviruses* and Henipa-like Viruses

The clade of shrew/rodent-borne *Parahenipaviruses* and henipa-like viruses appear to be distinct in tropism compared to the bat-borne *Henipaviruses*. Structural studies of the MojV and LayV G proteins indicate that their receptor-binding sites are incompatible with binding ephrin-B1, -B2, and -B3 [69,70]. Biochemical studies further confirmed that MojV and LayV G proteins do not bind to ephrins [71,72]. Thus, the entry receptor(s) for this group of viruses is yet to be determined.

Similarly, little is known about the role of endocytosis in the trafficking and processing of F proteins from rodent- or shrew-borne viruses. A sequence alignment of all HNV F protein cytoplasmic tails shows that they contain one or more Tyr residues (Figure 4, Tyr residues highlighted red). Of the rodent- or shrew-borne viruses, only CHV has a canonical endocytic motif of the form YXXØ (Figure 4, YSRI) and none encode a di-Tyr motif close to the C-terminus as observed for most of the bat-borne *Henipaviruses*. However, some have a single Tyr residue close to the end of the CT. It remains to be experimentally determined if any of the non-canonical, Tyr-based endocytic signals are functional. Preliminary data from our lab indicate that mutating the putative, Tyr-based endocytic signal of MojV F (Y530A) results in F protein accumulation in the cell. This is the same phenotype observed with the corresponding endocytic motif mutants of HeV and CedV F proteins [23,57]. Further experiments are needed to determine if the MojV F-Y530A mutation disrupts F endocytic trafficking and if this impacts cleavage. Similarly, experiments are needed to determine if any of the putative Tyr-based endocytic signals in the cytoplasmic tails of *Parahenipaviruses* and other unclassified henipa-like viruses are functional.

It is not known which proteases cleave *Parahenipavirus* and henipa-like virus F proteins to generate the fusion-active form. Studies investigating the proteolytic cleavage of MojV and LayV F in mouse Neuro-2a cells showed that treatment with the general cysteine protease inhibitor E-64d had no effect on the ratio of cleaved-to-uncleaved F proteins [70]. Thus, cathepsin L or B do not cleave these F proteins. Interestingly, all the known or predicted (based on sequence alignment) cleavage sites for the HNV F proteins are compatible with proteolytic cleavage by endosomal cathepsins (Figure 5).

In summary, very little is known about the role of endocytic trafficking and the identity of the cellular proteases that cleave *Parahenipaviruses* and henipa-like virus F proteins. Limited experiments with the closely related shrew-borne LayV and the rodent-borne MojV F proteins point to a different activation pathway compared to the bat-borne *Henipaviruses* [70]. Whether this holds true for all the other *Parahenipaviruses* and henipa-like viruses is yet to be determined.

## 6. Role of the Fusion Protein in Particle Assembly and Budding

For most paramyxoviruses, M is the principal driver of particle assembly and budding [73]; for HNVs three proteins, M, F, and even the attachment (G) protein can alone induce the formation of virus-like particles (VLPs) [74]. NiV M and F efficiently bud autonomously, while G does so with much lower efficiency [75]. Similarly, HeV F can also bud alone, but studies show that the M and F proteins coordinate with each other to form VLPs that are morphologically and physically distinct from either M-only or F-only VLPs [76].

F protein endocytic trafficking is required for proper assembly of virus particles and their budding [76,77]. The HeV F S490A mutant, which is endocytosis-defective and largely retained at the cell surface with little cleavage, lost both its intrinsic VLP release function and its ability to assemble with M for VLP production [76]. Thus, mutations in the HeV F transmembrane domain or cytoplasmic tail that disrupt endocytic trafficking led to the failure of F to interact with M during VLP assembly. For NiV, mutations or deletions in the YXXØ (YSRL) endocytic motif and the di-Tyr motif significantly reduced budding efficiency [75]. NiV F-driven VLP formation (either alone or with M and G) relies heavily on vesicular trafficking and actin cytoskeletal processes [77]. NiV F on biological membranes forms distinctive nanoclusters that are independent of F cleavage and are maintained by NiV F–AP-2 interactions [54]. Moreover, NiV F-driven VLP formation enhanced the recruitment of G into VLPs, implicating F in recruiting G into virus particles. In support of this, mutagenesis of the CedV F endocytic motif resulted in virus particles with significantly lower G incorporation.

Blocking NiV F maturation using the E-64d cysteine protease inhibitor did not impact the nanoscale organization of the F protein into clusters at the cell surface: F clusters formed by E-64d-treated uncleaved F_0_, or those formed by a mixture of F_0_ and cleaved F_1+2_ in untreated control cells, are similar [54]. Since both F_0_ and F_1+2_ co-exist in the same cluster on the cell membrane, they may also co-exist in released virus particles (Figure 1a). Similarly, when HeV F cleavage was blocked by E-64d [76], the wild-type F protein could still be incorporated into VLPs and function normally for VLP assembly with M, indicating that it is the endocytic trafficking pathway of F that is important for assembly, not necessarily the cleavage event itself. On the other hand, mutating the HeV F cytoplasmic tail endocytic motif (Y525A), which significantly reduced the rate of F endocytosis and led to two times more F at the cell surface than wild-type F, did not impact VLP formation nor M incorporation into VLPs [76]. This indicates that even a low level of F endocytic trafficking is sufficient for VLP formation and, by extension, virus particle assembly.

It was also shown that HeV F and M traffic through Rab11-positive recycling endosomes, and budding is at least partially dependent on Rab11 activity [76]. Together, these data indicate that the endocytic trafficking of the F protein is required for efficient particle assembly, possibly by allowing F to intersect with the M protein-trafficking pathway, potentially in Rab11-positive recycling endosomes. F may also facilitate the incorporation of the G protein into viral particles. We do not yet know where F intersects with G but given the role of endocytic trafficking in the assembly process, it may be in an endosomal compartment.

Finally, little is known about the role of F trafficking in the assembly of the other *Henipaviruses* (AngV, GhV, and CedV), but given that they all contain canonical YXXØ endocytic motifs (Figure 4), we can expect similarities with HeV and NiV assembly mechanisms, with some species-specific variations. As for the *Parahenipavirus* and other unclassified henipa-like virus F proteins, even less is known. While they all contain putative Tyr-based endocytic signals, only CHV encodes a canonical endocytic motif (Figure 4, CHV, YSRI). Further investigation into the functional role of these signals in endocytosis and F processing will yield important insight into the role of the F protein in the assembly and budding of rodent- and shrew-borne viruses.

In summary, for *Henipaviruses* like NiV and HeV, the F protein plays a more direct and significant role in particle assembly and budding than for the F proteins of other paramyxoviruses. *Henipavirus* F proteins’ unique endocytic trafficking pathway is crucial for efficient interactions with M and appears to enhance the incorporation of the G protein into viral particles. Specific motifs in the F cytoplasmic tail facilitate efficient budding, potentially by interacting with host cellular machinery involved in vesicular trafficking and the actin cytoskeleton.

## 7. Future Perspective

Beyond the role of F trafficking in proteolytic cleavage and particle assembly, the mainly endosomal localization of the F protein may also impact the host immune response. Sequestering the F protein away from the plasma membrane would partially hide it from immune surveillance. In this context, the innate and adaptive immune responses mounted against mutant viruses with slower F endocytic rates should be characterized. Furthermore, structural and biochemical analysis of virus particles and VLPs from such viruses would give insights into the mechanisms of particle assembly and budding. These studies may also inform vaccine design, as perturbations in F endocytic rates may increase immune recognition and reduce pathogenesis.

## Figures and Tables

**Table 1:** Alphabetical list of *Henipaviruses*, *Parahenipaviruses*, and henipa-like viruses.

Virus Name ^1^	Genus/Status ^2^	Natural Reservoir	Geographic Location	Year	F Protein Accession #
Angavokely virus (AngV)	*Henipavirus*	Madagascar fruit bats	Madagascar (Angavokely Cave)	2019	UVG43988.1
Camp Hill virus	Henipa-like virus	Northern short-tailed shrews	USA (Alabama)	2025	XJU75810.1
**Cedar virus (CedV)**	*Henipavirus*	Bats (*Pteropus* spp.)	Australia (Cedar Grove, Queensland)	2009	AFP87278.1
Daeryong virus (DARV)	*Parahenipavirus*	Shrews (*Crocidura lasiura* and *Crocidura shantungensis*)	Republic of Korea	2018	QYO90531.1
Chodsigoa hypsibia henipavirus	Henipa-like virus	Shrew (*Chodsigoa hypsibia*)	China	2022	WEU70826.1
Crocidura tanakae henipavirus	Henipa-like virus	Shrew (*Crocidura lasiura*)	China	2023	WZI33221.1
Denwin virus (DewV)	*Parahenipavirus*	Greater white-toothed shrews (*C. russula*)	Belgium	2022	WPS63725.1
**Gamak virus (GAKV)**	*Parahenipavirus*	Shrews *(Crocidura lasiura* and *Crocidura shantungensis)*	Republic of Korea	2018	QYO90517.1
Ghana virus (GhV)	*Henipavirus*	African straw-colored fruit bats (*Eidolon helvum*)	Ghana (Kumasi)	2008	AFH96010.1
Hasua virus (HasV)	Henipa-like virus ^3^	*Crocidura suaveolens* shrews	Germany (north-eastern)	2023	WPS63719.1
**Hendra virus (HeV)**	*Henipavirus*	Bats (*Pteropus* spp.)	Australia (Hendra, Brisbane).	1994	NP_047111.2
Jingmen Crocidura shantungensis henipavirus 1	*Parahenipavirus*	Shrews (*Crocidura shantungensis*)	China (Jingmen)	2021	UOX72983.1
Jingmen Crocidura shantungensis henipavirus 2	Henipa-like virus	Shrews (*Crocidura shantungensis*)	China (Jingmen)	2021	UOX72990.1
**Langya virus (LayV)**	*Parahenipavirus*	Shrews (*Crocidura* spp.)	China (Eastern China)	2022	UUV47205.1
Lechcodon virus (LechV)	Henipa-like virus ^3^	Shrews (*Crocidura leucodon*)	Germany (southern)	2023	WPS63705.1
Melian virus (MeliV)	*Parahenipavirus*	Large-headed forest shrews (*C. grandiceps*)	Guinea	2022	UQM99518.1
Mojiang virus (MojV)	*Parahenipavirus*	Cave rats (*Rattus flavipectus*)	China (Mòjiāng Hani Autonomous County, Yunnan Province)	2012	YP_009094094.1
Ninorex virus (NinExV)	*Parahenipavirus*	Eurasian pygmy shrews (*Sorex minutus*)	Belgium	2022	WJL29504.1
**Nipah virus (NiV)**	*Henipavirus*	Bats (*Pteropus* spp.)	Malaysia (Peninsular Malaysia)	1998	NP_112026.1
Peixe-Boi virus (PBV)	Henipa-like virus	Brazilian opossum (*Marmosa demerarae*)	Brazil	2015	Not available
Resua virus (ResV)	Henipa-like virus ^3^	Shrews (*Crocidura suaveolens*)	Germany (north-eastern)	2023	WPS63698.1
Shiyan Crocidura tanakae henipavirus (SCtV)	Henipa-like virus	Taiwanese gray shrews (*C. tanakae*)	China (Taiwan, Shiyan)	2024	WIU81496.1
Wenzhou Apodemus agrarius henipavirus 1	*Parahenipavirus*	Striped field mouse (*Apodemus agrarius*)	China (Wenzhou)	2021	UBB42272.1
Wenzhou shrew henipavirus 1	Henipa-like virus	Shrew	China (Wenzhou, Zhejiang)	2023	WPV62326.1
Wufeng Crocidura attenuata henipavirus 1	*Parahenipavirus*	Shrews (*Crocidura attenuate*)	China (Wufeng)	2021	UOX73004.1
Wufeng Chodsigoa smithii henipavirus 1	*Parahenipavirus*	Shrews (*Chodsigoa smithii*)	China (Wufeng)	2022	UOX72997.1

^1^ Viruses that have been isolated are indicated in bold font. All other viruses are inferred from complete or partial genetic sequences. ^2^ The genus was assigned based on ICTV classification at time of publication. Unassigned viruses are denoted as henipa-like viruses. ^3^ The authors classified these as *Orthoparamyxoviruses* but for clarity, we refer to them in this review as henipa-like viruses.

## Abbreviations

The following abbreviations are used in this manuscript:

AngV	Angavokely virus
CedV	Cedar virus
CFR	case fatality rate
CHV	Camp Hill virus
ChyV	Chodsigoa hypsibia henipavirus
CT	cytoplasmic tail
CtV	Crocidura tanakae henipavirus
DARV	Daeryong virus
DewV	Denwin virus
FP	fusion peptide
GAKV	Gamak virus
GhV	Ghana virus
HasV	Hasua virus
HeV	Hendra virus
HNV	henipavirus
HRA	heptad repeat A
HRB	heptad repeat B
ICTV	International Committee on Taxonomy of Viruses
JCS-1	Jingmen Crocidura shantungensis henipavirus 1
JCS-2	Jingmen Crocidura shantungensis henipavirus 2
LayV	Langya virus
LechV	Lechcodon virus
MEFs	mouse embryonic fibroblasts
MelV	Melian virus
MojV	Mojiang virus
NinExV	Ninorex virus
NiV	Nipah virus
ResV	Resua virus
RNP	ribonucleocapsid
SCtV	Shiyan Crocidura tanakae henipavirus
shRNA	small hairpin RNA
TM	transmembrane
VLP	virus-like paticle
WAa-1	Wenzhou Apodemus agrarius henipavirus 1
Wca-1	Wufeng Crocidura attenuata henipavirus 1
WCs-1	Wufeng Chodsigoa smithii henipavirus 1
WsV1	Wenzhou shrew henipavirus 1
